# Metronidazole Encephalopathy EEG Features: A Case Report with Systematic Review of the Literature

**DOI:** 10.3390/brainsci10040227

**Published:** 2020-04-10

**Authors:** Lorenzo Ricci, Francesco Motolese, Mario Tombini, Jacopo Lanzone, Roberta Rea, Francesco Di Matteo, Vincenzo Di Lazzaro, Giovanni Assenza

**Affiliations:** 1 Unit of Neurology, Neurophysiology, Neurobiology, Department of Medicine, University Campus Bio-Medico of Rome, via Álvaro del Portillo 21, 00128 Rome, Italy; m.tombini@unicampus.it (M.T.); j.lanzone@unicampus.it (J.L.); v.dilazzaro@unicampus.it (V.D.L.); g.assenza@unicampus.it (G.A.); 2 Digestive Endoscopy Unit, Department of Medicine, University Campus Bio-Medico of Rome, via Álvaro del Portillo21, 00128 Rome, Italy; r.rea@unicampus.it (R.R.); f.dimatteo@unicampus.it (F.D.M.)

**Keywords:** metronidazole-induced encephalopathy, MIE, metronidazole, drug-induced seizure, EEG

## Abstract

Metronidazole-induced encephalopathy (MIE) is a rare and often under-recognized iatrogenic condition. The diagnosis should be considered in metronidazole-treated patients presenting with acute encephalopathy, unprovoked seizures and cerebellar signs. While typical magnetic resonance imaging (MRI) findings strongly support the diagnosis, electroencephalography (EEG) features have been rarely reported and poorly described. We present a longitudinal EEG assessment in one patient with encephalopathy due to metronidazole toxicity who presented a peculiar EEG pattern presentation and evolution. During the acute phase of encephalopathy, the EEG showed a monomorphic, sharply contoured theta activity symmetrically represented over frontal regions with an anterior–posterior progression which evolved in parallel with clinical worsening. Together with a systematic review of the literature, we discuss whether this EEG activity may represent a distinct neurophysiological correlate of ‘cerebellar encephalopathy’.

## 1. Introduction

Metronidazole is an antimicrobial agent commonly used in the treatment of several protozoal and anaerobic infections. It is usually well tolerated, but it has occasionally been associated with the development of serious neurological adverse events, including peripheral neuropathy, cerebellar dysfunction, visual impairment, vestibulotoxicity, cochleotoxicity, ataxic gait, dysarthria, seizures and encephalopathy (metronidazole-induced encephalopathy, MIE) [1]. The incidence is undetermined and case reports suggest a peak in the sixth and seventh decades of life [2]. MIE is commonly reversible on discontinuation of metronidazole, but long-lasting sequelae and even fatal outcomes have been reported [3]. The exact dose required to provoke MIE is unknown since toxicity has been reported either at low or at high doses [4,5]. For these reasons, it is advisable to find diagnostic tools allowing a prompt diagnosis soon after symptoms onset. In this scenario, despite the wide spectrum of clinical manifestations, MIE is supported by specific magnetic resonance imaging (MRI) findings. The most reliable feature is a symmetrical T2-weighted or fluid-attenuated inversion recovery (FLAIR) hyperintensity and minimal T1-weighted hypointensity of cerebellar dentate nuclei [6], which could reverse after drug discontinuation [3]. Despite the frequent impairment of consciousness, it is surprising that electroencephalography (EEG) features of patients with MIE have been rarely reported in the literature. Furthermore, they are scarcely documented and lack any qualitative and topographic characterization. Herein, we provide a case report of one patient with MIE and his detailed longitudinal EEG monitoring, which revealed a peculiar pattern of presentation and evolution. Therefore, we performed a systematic review of the literature searching for EEG descriptions in patients with MIE with the aim of understanding whether MIE shows specific EEG changes, which could help expedite the appropriate diagnosis of this rare condition.

## 2. Case Presentation

**Figure 1:**
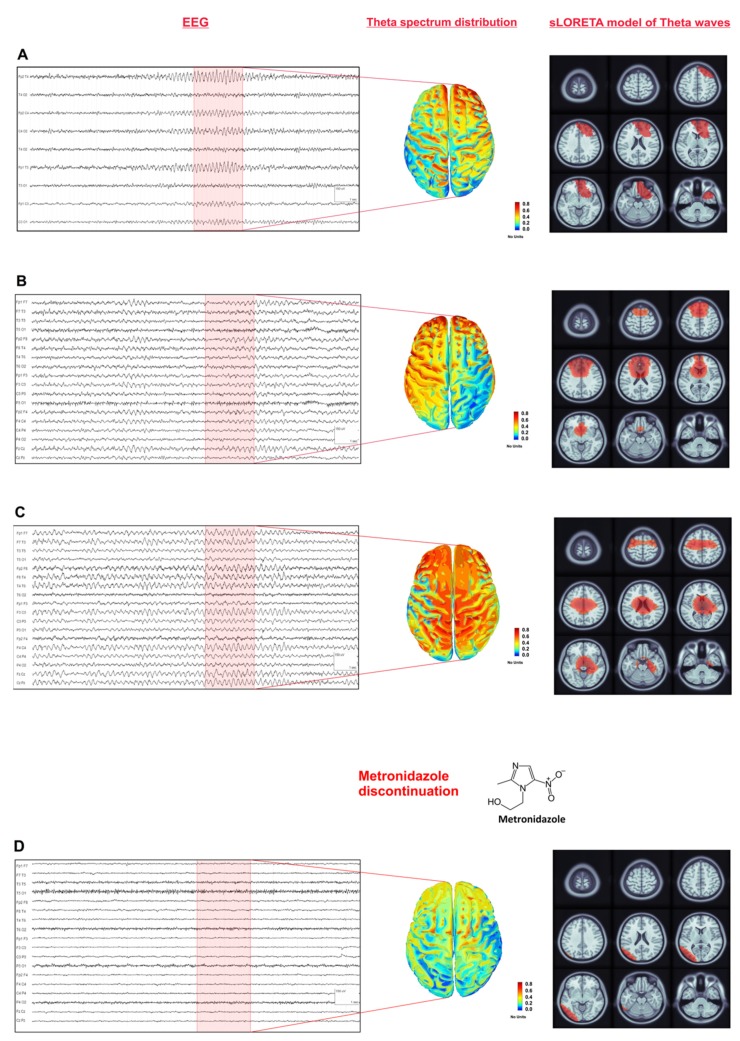
Standard 19-channel electroencephalography (EEG) recordings of our patient (high pass filter: 1.6 Hz; low pass filter: 50 Hz; left panel), standardized low-resolution brain electromagnetic tomography (sLORETA) distributed solution of normalized relative theta (5–7 Hz) power density at the cortical voxels (middle panel) and source analysis of averaged theta waves (right panel). LORETA computes 3D linear solutions for the EEG inverse problem within a 3-shell spherical head model including scalp, skull and brain compartments. (**A**) shows the EEG obtained after our first examination characterized by frequent and very brief runs of bilateral and symmetric monomorphic sharp theta activity over frontal regions. Serial EEGs, performed 8 (**B**) and 12 days (**C**) later, confirmed the presence of rhythmic monomorphic sharp theta activity, organized in more prolonged sequences. sLORETA distributed solution for theta frequency power spectrum confirms the anterior distribution of slow activity. sLORETA source analysis of averaged theta waves shows an anterior distribution of electrical generators for the first 2 EEGs as well (**A**,**B**); while the last EEG displays deeper electrical generators (bilateral thalami and subcortical white matter, (**C**)) which may suggest the involvement of dentate-thalamo-cortical tracts. Values in this figure represent the relative power of each source. At each vertex of the cortex surface, the value between 0 and 1 indicates the contribution of the current frequency band to the total power in the signal. Panel (**D**) shows a 1-month follow-up EEG. No abnormalities are noticed. sLORETA solution shows normalization of the theta-band anterior distribution as well. sLORETA solution was performed using the Brainstorm toolbox for Matlab and age-appropriate head templates [7]. The left side of cortical images and 2D magnetic resonance imaging (MRI) corresponds to the left hemisphere.

A 64-year-old Caucasian man came to our attention because of bilateral tonic-clonic seizures followed by altered mental status. He was hospitalized for three months because of an intra-abdominal abscess which he developed after jejunal perforation related to endoscopic retrograde cholangiopancreatography (ERCP). No history of alcohol intake or substance abuse was reported. He had been receiving metronidazole 500 mg four times per day for about 10 weeks (cumulative dose: 126 g). On examination, he was alert but confused and unable to perform either complex or simple motor tasks. Spontaneous speech was minimal, and no ocular abnormalities were noted except for bilateral nystagmus on extreme gaze. Slight dysmetria in his upper limbs was observed and he referred numbness in hands and feet. During hospitalization, he also experienced some episodes of psycho-motor agitation and, on other occasions, transitory alteration of consciousness with unresponsiveness that raised the suspicion of focal seizures with impaired awareness. An EEG was performed, showing frequent and very brief (<10s) runs of waxing and waning, bilateral and symmetric medium voltage monomorphic sharp theta activity over fronto-central regions (Figure 1A).

**Figure 2:**
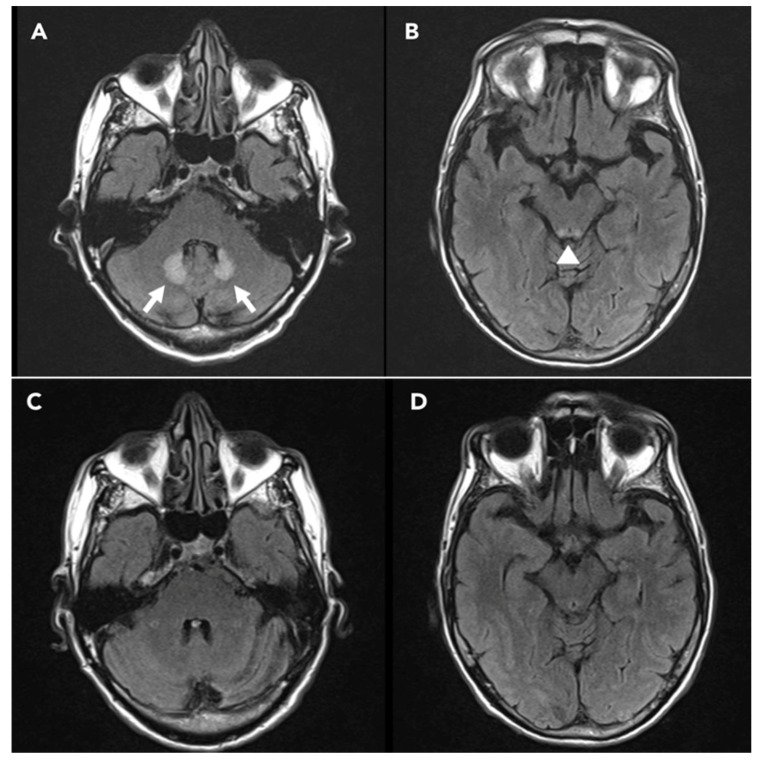
A 1.5 Tesla brain MRI. Fluid-attenuated inversion recovery (FLAIR) sequences showing hyperintensity in (**A**) bilateral dentate nuclei of the cerebellum (arrows) and (**B**) dorsal midbrain (arrowhead). A follow-up MRI performed one month after metronidazole discontinuation did not show any abnormalities (**C**,**D**).

Levetiracetam 250 mg twice a day was started. A few days later, he presented a new episode of impaired awareness followed by focal-to-bilateral tonic-clonic seizure, therefore levetiracetam was increased up to 500 mg twice a day. After the occurrence of a new cluster of rapidly occurring bilateral tonic-clonic seizures, the patient received lorazepam 4 mg i.v. and levetiracetam 1000 mg i.v. A brain MRI showed enhanced FLAIR signal bilaterally in the dentate nuclei of the cerebellum and in the midbrain tectum, with diffusion restriction and without contrast enhancement (Figure 2A,B).

Three days later, his mental status was still partially altered. EEG performed 5 and 6 days after the first tonic-clonic seizure confirmed the persistence of rhythmic monomorphic sharp theta activity, organized in more prolonged sequences symmetrically involving fronto-centro-temporal regions (Figure 1B,C). Administration of diazepam (10 mg i.v.) did not induce any clinical or EEG improvement, thus making unlikely a diagnosis of non-convulsive status epilepticus (NCSE) [8]. Cerebrospinal fluid examination showed no signs of central nervous system inflammation and polymerase chain reaction for neurotropic viruses was negative; no electrolyte shifts were noticed. Typical brain MRI findings in association with acute encephalopathy after a very prolonged course of metronidazole therapy led to a clinical diagnosis of MIE. Metronidazole was stopped and the patient’s clinical conditions improved during the next couple of weeks. No more seizures occurred and, 20 days after the first convulsive episode, he presented a complete recovery of his mental status. At the 1-month follow-up examination, EEG and brain MRI abnormalities were no longer evident (Figure 1D and Figure 2C,D, respectively).

## 3. Systematic Review

A systematic review was performed applying the PRISMA (Preferred Reporting Items for Systematic Reviews and Meta-Analyses) guidelines [9]. Full-text articles were selected from a comprehensive search of PubMed, Medline, Scopus and Google Scholar databases. Keywords and their synonyms were combined in each database as follows: (“metronidazole”) AND (“intoxication” OR “toxicity” OR “encephalopathy” OR “epilepsy” OR “seizure” OR “EEG”). No filter was applied on the publication date of the articles, and all results of each database were included up to January 2020. After the removal of duplicates, all articles were evaluated through a screening of titles and abstracts by three independent reviewers (L.R., F.M., G.A.). The same three reviewers performed an accurate reading of all full-text articles assessed for eligibility to this study and they performed a collection of data to minimize the risk of bias. In case of disagreement among the investigators regarding the inclusion and exclusion criteria, the senior investigator (G.A.) made the final decision.

Articles were included if they met the following inclusion criteria: (i) described patients with neurological signs or symptoms attributed to metronidazole toxicity (e.g., seizures or impairment of consciousness); (ii) included a description or a picture of at least one EEG recording; (iii) written in English language; and (iv) published in a peer-reviewed journal.

The exclusion criteria were: (i) the study reported patients with more than one apparent cause of encephalopathy; (ii) studies conducted in animals or in vitro models; and (iii) conference proceedings, reviews and books.

### 3.1. Data Extraction Process

**Figure 3:**
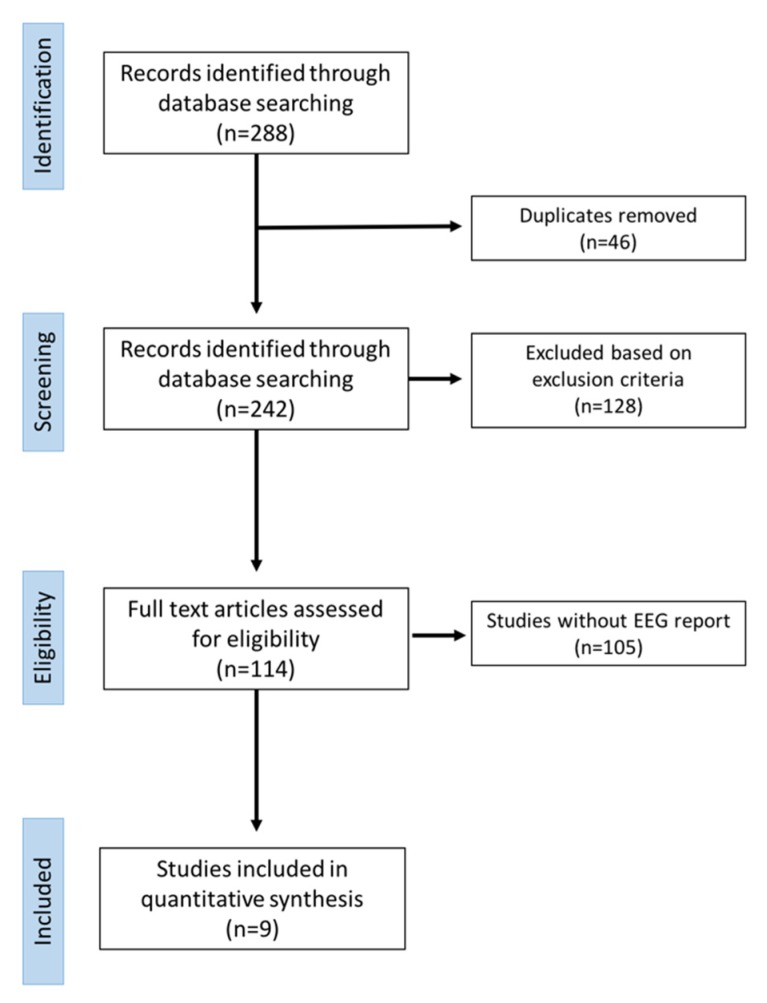
PRISMA flowchart of the selection of the studies for this review.

Data extraction was executed on 288 articles (Figure 3).

Fourty-six articles were excluded because of duplicates. Data were extracted on the basis of the following checklist: authors, year and type of publication (i.e., case report, review); characteristics of the participants involved in the study and aim of the study; and presence of EEG description (yes or no).

After an accurate revision of full manuscripts, nine articles satisfied the inclusion/exclusion criteria (Table 1).

### 3.2. Results

We identified nine cases of MIE with an EEG description; however, EEG recordings were only available in three cases for critical revision. Most cases presented normal or unspecific EEG findings and the majority of seizures, if present, were reversible after the discontinuation of metronidazole. In one case only, NCSE was the clinical manifestation of metronidazole toxicity [14]. In two cases the EEG pattern included generalized periodic or quasi-periodic discharges (GPDs) with triphasic morphology [14,17]. In one case, focal left anterior fronto-central slowing and interictal sharp waves in the same regions were described [15]. In the remaining cases, EEG findings described “diffuse slowing” without epileptic discharges and with a prompt resolution after metronidazole discontinuation [10,11,13,16] or normal findings [12,18] (see Table 1).

Finally, our systematic review suggests that the EEG findings in MIE do not seem to correlate with clinical outcome, with the presence of seizures, with the severity of encephalopathy, nor with the cumulative dose of metronidazole therapy.

## 4. Discussion

MIE is an uncommon complication of metronidazole therapy. It typically manifests with dysarthria and gait instability, but altered mental status, convulsive seizures and permanent sequelae have been reported [3]. Risk factors include liver dysfunction and long-lasting therapy with metronidazole (typical cumulative dose: > 20 g) [3].

Bilateral tonic-clonic seizures are an uncommon manifestation of MIE and the EEG findings associated with this condition are considered unspecific [19]. In general, EEG has been successfully applied to identify epileptic states or interictal pattern or whether an altered mental status derives from lateralized focal dysfunction or significant metabolic alterations; however, the correlation between EEG patterns, imaging findings and specific clinical diagnoses such as MIE are underrecognized, and much of our understanding of these correlations come from isolated case reports [20]. Investigations with electro-clinical-neuroimaging correlations would expedite appropriate diagnosis and clinical management of patients in the ICU and neurological ward, improving patients’ care and shortening the duration of patients’ stay in the hospital [20,21].

We observed a very characteristic EEG pattern in our patient (Figure 1), displaying a peculiar evolving trend. Such EEG features were characterized by the initial appearance of sporadic sharp theta activity over the anterior regions, which ultimately progressed to a rhythmic, quasi-continuous, medium voltage and monomorphic sharp theta activity involving the whole brain, with an anterior–posterior gradient and without clinical or electrographic modifications after the infusion of benzodiazepines.

The systematic review of the literature did not highlight a specific EEG pattern for MIE, neither did it confirm our own findings, although in one case, the focal slowing was mainly anterior [15]. However, it is worth mentioning that most of the reviewed cases lacked EEG recordings for critical revision, while only brief descriptive comments were provided (e.g., “diffuse slowing”). Most patients did not perform a longitudinal EEG assessment coupled with clinical worsening, as in our case. Such missing information may lead to imprecise estimates of the prevalence of typical EEG features.

The possible pathogenic mechanisms and the neurophysiological explanations for the anterior predominance and diffusion of the EEG slow abnormalities in MIE could be diverse.

The influence of cerebellum, which is one of the main targets of metronidazole toxicity [3], on frontal cognition and excitability is quite well described. Middleton and Strick discovered in the 1990s that the deep cerebellar nuclei direct information to prefrontal areas through dentato-thalamic pathways, while the prefrontal cortex sends information back to the cerebellum via pontine nuclei [22]. Moreover, studies of adult patients with acquired cerebellar lesions provided evidence for disruption of selective attention, such as orienting, distributing and shifting attention [23,24], and in patients with subtentorial damage undergoing surgery, it is well-described as the so-called “posterior fossa syndrome”, which is characterized by a cerebellar mutism caused by dentato-thalamo-cortical pathway dysfunction [25,26].

These anatomo-clinical correlations support our own findings showing a frontal EEG prevalence of the toxic insult during the early phase of MIE. Moreover, sLORETA solution performed on our patient’s EEG (see Figure 1) provides interesting insights: theta frequency spectrum increased throughout time, mostly over the anterior regions at scalp level, corresponding to the EEG visual alterations. Source analysis revealed that the electrical generators of theta waves were initially bilaterally localized in the cortical-subcortical frontal regions; however, the last EEG which displayed the most severe spread of theta activity over the whole brain, revealed deep electrical generators involving bilateral thalami and subcortical white matter. We may speculate that such localization results provide clues regarding the involvement of dentate-thalamo-cortical tracts during the final stages of MIE, thus justifying the anterior prevalence of theta abnormalities in these patients. However, it is important to emphasize that electrical source imaging methods (i.e., sLORETA) are undetermined; thus, the images are estimates. Error can be caused by uncertainty of the exact conductivity of brain tissue, skull and scalp [27]. Besides, electrical source imaging with low-density EEG is less accurate than high-density EEG (128–256 channels) source imaging [28,29]. Further assessments are needed to better confirm the correlation among EEG, MRI, electrical source imaging and metronidazole toxicity in large case series and prospective studies, to increase the value of EEG in encephalopathy and improve clinical management in such patients.

In our patient, the acute encephalopathy was evident in the EEG through a rhythmic oscillatory activity in the theta frequency range. Neural oscillations underlie brain function and are essential to ensure complex and integrative tasks for both cerebral and cerebellar cortex [30]. Previous studies have shown that cerebellar oscillations may interact with cerebral oscillations in humans [31], possibly to relate cerebellar activity with distant cerebral areas [32].

The increase in theta activity is usually caused by an alteration of the underlying brain tissue or its network [33], and specific theta/beta (4–25 Hz) oscillations of the granule cell layer in the cerebellum have been linked to cerebral cortex activity [32,34]. Di Lazzaro and colleagues have shown that cerebellar functional lesions produced by transcranial magnetic stimulation (TMS) produce an increase in theta EEG activity in frontal areas, suggesting a direct link between cerebellar dysfunction and increased EEG theta frontal activity [35].

In turn, the frontal disconnection from the cerebellum may cause an increase in cortical excitability and thus explain an increased risk of seizures [35]. In fact, the inhibitory role of the cerebellum on cerebral cortex is well known and its invasive stimulation is exploited for palliative epilepsy surgery in drug-resistant epilepsy patients [36,37]. Conversely, cerebellar lesions can result in various types of epileptic seizures, including bilateral tonic-clonic seizures, which disappear after complete resection of the affected region [38]. This could explain the occurrence of seizures in MIE patients, as in our patient.

## 5. Conclusions

In conclusion, we provide a prospective longitudinal EEG study of one patient with MIE showing a peculiar EEG pattern evolution. The systematic review of the literature does not support an EEG peculiarity in these patients, because of a substantial lack of accurate reports and longitudinal assessments. However, our data suggest a possible specific EEG pattern (sharp theta activity with a progressive antero-posterior diffusion), which could fit with the radiological and clinical evidence of cerebellar dysfunction caused by MIE.

Clinicians should be aware of this possible EEG manifestation of MIE since it may eventually suggest the diagnosis of this rare condition, which requires the prompt withdrawal of metronidazole therapy in order to prevent severe and permanent neurological sequelae.

## Figures and Tables

**Table 1:** Review of EEG findings and clinical outcomes of MIE case reports.

Author, Year	Age (y), Sex	Cumulative dose of Metronidazole (grams)	Neurological Presentation	MRI Findings	EEG Findings	EEG Follow up Findings	Neurological Outcome
**Bailes et al. 1983 [10]**	12, M	4	Focal to bilateral tonic clonic seizures.	Not performed	Diffuse slowing without epileptic discharges (**NOT SHOWN**)	Normal	Resolution
**Beloosesky et al. 2000 [11]**	87, F	18	Focal to bilateral tonic clonic seizures.	Not performed	Diffuse slowing without epileptic discharges (**NOT SHOWN**)	Normal	Resolution
**Ferroir et al. 2009 [12]**	76, M	45	Peripheral Neuropathy, Cerebellar syndrome, focal to bilateral tonic clonic seizures.	T2 FLAIR hyperintensity in bilateral dentate nucleus, thalamus, splenium of corpus callosum.	Normal (**NOT SHOWN**)	Not done	Resolution
**Groothoff et al. 2010 [13]**	38, F	132	Cerebellar syndrome, altered mental status, focal to bilateral tonic clonic seizures.	Abnormal signal in T2 FLAIR in the centrum semiovale and cerebellar peduncles.	Unspecific encephalopathy findings (**NOT SHOWN**)	Unchanged	Death
**Cantador et al. 2013 [14]**	56, M	5	NCSE †	Hyperintense lesion of the dentate nucleus, middle cerebellar peduncles, olivary nuclei and corpus callosum in FLAIR sequences.	GPDs ‡	Normalization	Resolution
**Hobbs et al,.2015 [15]**	65, F	33	Confusion and disorientation.	Symmetrical T2 hyperintensity and generally restricted diffusion in bilateral dentate nuclei, corpus callosum, midbrain, superior cerebellar peduncles, internal capsules, and cerebral white matter.	Diffuse slowing, focal left fronto-central slowing, epileptiform sharp waves at left fronto-central (**NOT SHOWN**)	Unchanged	Coma and Death
**Önder. 2016 [16]**	68, F	10.5	Confusion and disorientation.	MRI showing bilateral globus pallidus T1 hyperintensities.	Diffuse slowing in theta-delta rhythm	Normalization	Resolution
**Wang et al. 2017 [17]**	58, F	94.5	Rapidly progressive consciousness disturbance with vegetative state.	Abnormal high signals in T2 FLAIR at bilateral dentate nuclei of the cerebellum, midbrain and dorsal pons and restricted diffusion in bilateral periventricular white matter, anterior and posterior splenium of corpus callosum.	Generalized high-voltage sharp wave complexes in quasi-periodic patterns with TM **.	Slow background activity at theta range	Unchanged
**Sørensen et al. 2018 [18]**	66, F	78	Cerebellar syndrome, focal to bilateral TC seizures, multifocal myoclonus.	Abnormal T2 FLAIR hyperintense signal changes in the bilateral dentate nuclei.	Normal (**NOT SHOWN**)	Not done	Resolution

†, Non-convulsive status epilepticus; ‡, generalized periodic discharges; **, triphasic morphology.
